# Hopping intermittent contact-scanning electrochemical microscopy (HIC-SECM) as a new local dissolution kinetic probe: application to salicylic acid dissolution in aqueous solution

**DOI:** 10.1039/c5ce00138b

**Published:** 2015-06-26

**Authors:** Amelia R. Perry, Robert A. Lazenby, Maria Adobes-Vidal, Massimo Peruffo, Kim McKelvey, Michael E. Snowden, Patrick R. Unwin

**Affiliations:** a Department of Chemistry, University of Warwick Gibbet Hill Road Coventry CV4 7AL UK p.r.unwin@warwick.ac.uk

## Abstract

Dissolution kinetics of the (110) face of salicylic acid in aqueous solution is determined by hopping intermittent contact-scanning electrochemical microscopy (HIC-SECM) using a 2.5 μm diameter platinum ultramicroelectrode (UME). The method operates by translating the probe UME towards the surface at a series of positions across the crystal and inducing dissolution *via* the reduction of protons to hydrogen, which titrates the weak acid and promotes the dissolution reaction, but only when the UME is close to the crystal. Most importantly, as dissolution is only briefly and transiently induced at each location, the initial dissolution kinetics of an as-grown single crystal surface can be measured, rather than a surface which has undergone significant dissolution (pitting), as in other techniques. Mass transport and kinetics in the system are modelled using finite element method simulations which allows dissolution rate constants to be evaluated. It is found that the kinetics of an ‘as-grown’ crystal are much slower than for a surface that has undergone partial bulk dissolution (mimicking conventional techniques), which can be attributed to a dramatic change in surface morphology as identified by atomic force microscopy (AFM). The ‘as-grown’ (110) surface presents extended terrace structures to the solution which evidently dissolve slowly, whereas a partially dissolved surface has extensive etch features and step sites which greatly enhance dissolution kinetics. This means that crystals such as salicylic acid will show time-dependent dissolution kinetics (fluxes) that are strongly dependent on crystal history, and this needs to be taken into account to fully understand dissolution.

## Abstract 2

Transiently induced dissolution of salicylic acid crystals reveals initial intrinsic dissolution kinetics.

## Graphical abstract

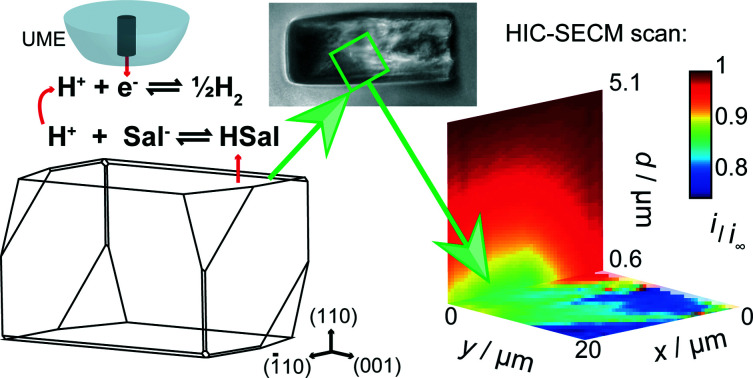

## Introduction

Crystalline substances, and their dissolution activity, are of wide-ranging interest, for example in natural processes,^1–4^ to understand and optimise construction materials^5,6^ and for foods and pharmaceuticals.^7–12^ This paper focuses on the dissolution of crystals of a model pharmaceutical, salicylic acid, which occurs naturally in willow bark^13^ and is a derivative of the widely-used painkiller, aspirin. In modern medicine it is used as a topical treatment for various skin ailments.^14^

In general, the kinetics of interfacial processes, such as dissolution, are controlled by two processes in series: diffusion of chemical species between the crystal surface and bulk solution, and the surface process itself,^15^ which may involve a myriad of interfacial phenomena. A process which is limited by the transport of species from the interface to bulk solution is referred to as being ‘diffusion’ or ‘mass transport’ controlled, whereas if mass transport between the surface and bulk solution is sufficiently high that the rate depends on surface kinetics, this is a ‘surface’ or ‘kinetic limited’ situation. Clearly, many processes will be under ‘mixed’ control, making it imperative that experimental techniques deliver well-defined mass transport.^15^ Additionally, dissolution processes are further complicated by the fact that crystal surfaces are microscopically complex which may impact the resulting kinetics and mechanism of dissolution.^15^

Among previous studies of salicylic acid dissolution, the use of a hydrodynamic flow cell combined with atomic force microscopy (AFM)^16–18^ is noteworthy as an attempt to study kinetics with controlled mass transport. In our recent work, we studied salicylic crystals with microscale dimensions and followed dissolution and growth using *in situ* optical microscopy, combined with finite element method (FEM) simulations.^9^ This produced detailed information regarding the kinetic behaviour of the crystals, particularly plane-dependent dissolution behaviour, but the system was predominantly under diffusion-control. Moreover, the long-duration of the measurements (as with most dissolution techniques) meant that the crystals were studied in an extensively reacted (heavily pitted) regime.

**Fig. 1:**
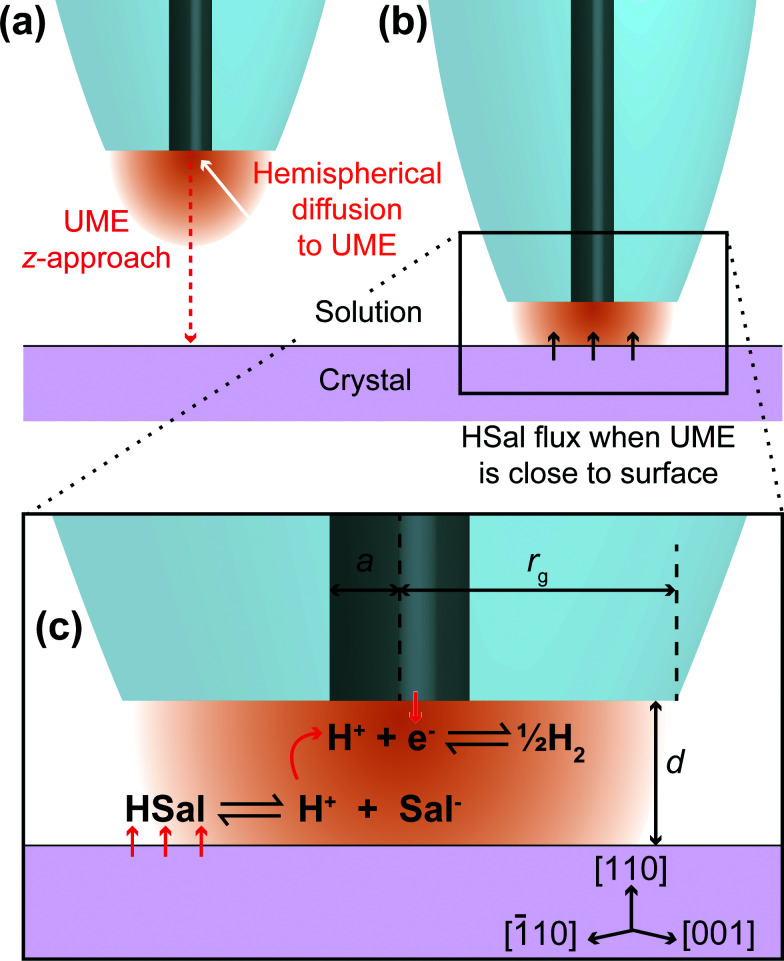
Schematic showing a UME in bulk solution, at a sufficient distance from the crystal that HSal/H^+^ reduction does not induce HSal dissolution (a) and close to the crystal such that the surface dissolves due to local undersaturation between the tip and crystal, induced by the UME process (b). (c) Zoom-in of part (b) showing the reactions occurring on the (110) face of salicylic acid and the UME. The reduction of protons at the UME surface causes the dissociation of salicylic acid through local undersaturation. When the UME is brought close to the salicylic acid crystal, this localised undersaturation causes the crystal surface to dissolve to replenish aqueous salicylic acid and is manifested as a higher current at the UME, than would be expected for an inert surface with the UME at the same distance. Note that this diagram is not to scale.

The present investigation uses scanning electrochemical microscopy (SECM)^19–21^ to study the dissolution of weak acid crystals, whereby a mobile, spatially controlled, ultramicroelectrode (UME) is used to induce dissolution locally and probe the resulting surface kinetics. There are many examples of this technique being applied to crystal dissolution in literature.^3,4,22–29^ The basic idea, as employed herein, is to use the UME to change the local solution concentration near the crystal/solution interface, to create an undersaturated solution. This leads to dissolution, and the chemical (dissolution) flux from the crystal is manifested in the measured tip current. In this study, the local salicylic acid concentration in solution is lowered (and controlled) by the reduction of protons at the electrode surface, as illustrated in Fig. 1. This causes the weak acid in solution to dissociate, thereby lowering the concentration of salicylic acid and causing an undersaturated solution. The approach developed herein greatly expands the range of systems that can be studied by SECM, but is also advantageous because the product (H_2_) is innocuous in the system, in contrast to some other earlier studies where the product may accumulate at the electrode,^23^ or where the product may impact dissolution.^15^ Here, it should be further noted that sodium salicylate is orders of magnitude more soluble than salicylic acid, and so although salicylate accumulates in the gap between the tip and substrate (and is present in bulk solution), the levels attained do not impact dissolution.

In this study, we utilise a recent development in SECM known as hopping intermittent contact (HIC)-SECM,^30^ which constructs a three-dimensional (3D) current map above an interface of interest,^30,31^ as well as the local substrate topography, from a series of vertical approaches of the tip to the substrate surface. The topographical map of the surface is obtained using the position where the UME makes intermittent contact (IC) with the surface. The advantage of this approach is that dissolution at each local position is only induced briefly (~1 s when the tip electrode is within a distance of a tip radius or so from the crystal surface) so that we measure the behaviour of an as-grown crystal (basal surface), in contrast to one which is heavily pitted (reacted), as in our previous study^9^ and other studies.^16,22^ Moreover, at each point across the surface, the current is measured effectively in bulk, *i*_∞_ (Fig. 1a) and close to the crystal, where dissolution is induced briefly, *i*_dis_ (Fig. 1b), and this provides a very sensitive measure (*i*_dis_/*i*_∞_) for dissolution, with the status of the electrode checked at every pixel across the crystal.

Herein, we will demonstrate that the analysis of experimental data with detailed finite element method (FEM) modelling of the tip electrode reaction and crystal process allows the range of dissolution fluxes at the crystal surface to be estimated. The dissolution kinetics for the (110) surface is at least an order of magnitude smaller than for a reacted surface, highlighting that, if the findings for salicylic acid are manifest in other pharmaceutical crystals, these materials will show dissolution kinetics – of importance in oral drug administration – hugely dependent on dissolution time and crystal history.

## Experimental

### Solutions, samples and electrodes

Microcrystals of salicylic acid were produced on poly-l-lysine (PLL) (molecular weight 70 000–150 000, highest purity available, Sigma-Aldrich) functionalised glass slides assembled into petri dishes as described recently by Perry *et al.*^9^ All solutions were prepared using ultrapure water (Milli-Q Reagent, Millipore) with a typical resistivity of 18.2 MΩ cm at 25 °C, and all chemicals were purchased from Sigma-Aldrich. For HIC-SECM measurements, 250 mM sodium salicylate (>99.5%) was combined in equal volumes with 10 mM sulphuric acid (>95%, Sigma) and stirred to mix thoroughly prior to scanning. These concentrations were chosen as they resulted in a solution with a salicylic acid concentration of close to 10 mM, as was determined by MINEQL^+^ (Environmental Research Software, version 4.6), which was just saturated and ensured that the crystal did not grow or dissolve noticeably during the timescale of a HIC-SECM scan (typically 45 min). This solution was filtered into the petri dish. For crystals imaged by AFM after dissolution driven by bulk undersaturation for a predetermined time, the concentration of salicylic acid was 8.4 mM, mimicking conditions used in our previous study.^9^

For voltammetry, approach curves and HIC-SECM, a two electrode setup was used with a 2.5 μm diameter platinum-disk UME serving as the working electrode that was fabricated in house.^32^ A saturated calomel electrode (SCE) was used as a quasi-reference counter electrode (QRCE). The UME was characterised by a ratio of the glass radius (*r*_g_) to platinum radius (*a*), known as the RG value (RG = *r*_g_/*a* in Fig. 1c),^33^ of about 15. Before measurements, the Pt UME was carefully polished using a moist microfibre pad (Buehler) covered with alumina suspension (0.05 μm particles, Buehler) in purified water. The UME was then rinsed and polished on a second microfibre pad containing only purified water, to remove any alumina.

### Instrumentation

The hardware used for imaging was a modified version of the recently reported setup for intermittent contact (IC)-SECM, and described in detail for HIC-SECM.^30^ The instrumentation differed in the fine control of the *x*, *y* and *z* position of the SECM tip, which was realised by a multi-axis nanopositioning system in closed loop operation with a 100 × 100 × 100 μm range (P-61135 NanoCube XYZ Piezo Stage, Physik Instrumente). This was mounted on an inverted optical microscope (Axiovert 25, Zeiss) with a 40× lens, used to visualise and locate suitable crystals for study. The tip electrode was directly mounted onto a piezo bender actuator (PICMA P-871.112, Physik Instrumente), which had a built in a strain gauge sensor (SGS), which measured the amplitude of the vertical oscillation that was applied to it. The piezo bender actuator reduced the force applied by the tip on the crystal, due to the lower spring constant, compared to other positioners.^34^

Steady-state cyclic voltammetry (CV) at the UME was performed in bulk solution to identify the potential required for the diffusion-limited reduction of protons (in the HSal solution) with respect to the SCE. This was −0.8 V *vs.* SCE, and was determined by the plateau of the voltammogram indicating a limiting current (*vide infra*).^35^ All imaging and CV measurements were controlled, and data acquired, using a LabVIEW 9.0 (National Instruments) program.

### HIC-SECM

A petri dish containing the salicylic acid crystals was placed on the inverted microscope, and a suitable crystal, 50–100 μm in the largest dimension, and isolated so that no other crystals were within a region of at least 40× the largest dimension, was located.

**Fig. 2:**
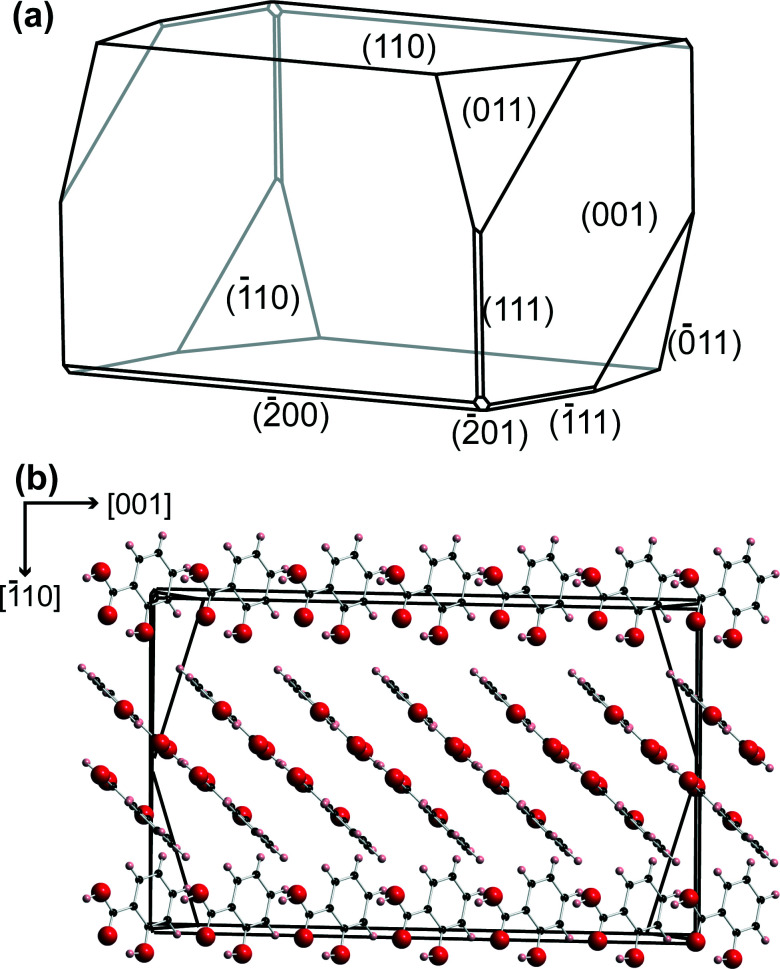
(a) Predicted morphology for salicylic acid using Mercury 3.3 software. (b) The relative orientation of the salicylic acid molecules in the crystal, perpendicular to the (110) plane.

Salicylic acid crystal results agreed with literature for the polymorph *P*2_1_/*a*.^36,37^ The (110) face of salicylic acid crystals was studied. A typical microcrystal is shown in Fig. 2, with the morphology and molecular arrangement predicted using Mercury software (Cambridge Crystallographic Data Centre, version 3.3). With the growth procedure used, the (110) surface was the top face, essentially in the same plane as the glass slide.^9^ The tip electrode was positioned above an appropriate salicylic acid crystal using course control of the *x*–*y* positioners, while the *z* position of the tip was also adjusted to be within 100 μm of the glass surface, using the optical microscope view as a guide.

Imaging was carried out using HIC-SECM, as described recently for other applications.^30^ A scan size of 20 × 20 μm in *x*–*y*, with a retraction in *z* position of 5 μm (distance of approach), was used. The HIC-SECM scan of crystal 1 consisted of 289 *z*-approaches (17 in both the *x*- and *y*-directions) whilst the scan of crystal 2 consisted of 400 *z*-approaches (20 in both *x* and *y*). Throughout a particular scan, the tip potential was held at −0.8 V, *i.e.* the potential required for the diffusion-limited reduction of H^+^/HSal, as determined by CV.

The tip was oscillated in *z* with a frequency of 80 Hz and a peak-to-peak amplitude of 37 nm. The tip was translated towards the crystal with a step size of 50 nm (spatial resolution of the current measurement) in the *z*-direction, at an overall tip velocity of ~0.5 μm s^−1^, to produce a *z*-approach curve. When the tip made physical contact with the crystal surface, the oscillation amplitude was damped. The amplitude setpoint for the damped amplitude was 30 nm, indicating IC, at which point the tip approach was terminated, and the tip was retracted in *z*, normal to the surface, and moved in *x*–*y* to the next point for a *z*-approach. The *z* position at the closest distance was stored and used to create a topography map. During each tip approach, the direct current (DC) at the working electrode was measured as a function of *z* to create 3D current maps which could be used for dissolution kinetic analysis.

### AFM of salicylic acid crystals

AFM was performed in order to visualise the surface morphology of the (110) face of salicylic acid crystals, as-grown (for the studies herein) and after dissolution, under conditions similar to our recent study.^9^ Topographical imaging of the crystal surfaces was carried out in air using tapping mode AFM (BioScope Catalyst with ScanAsyst, Veeco) with a Nanoscope V controller. The probe used was a sharp silicon nitride lever (SNL-10 A, Bruker). An optical image of the crystal was taken using a ×40 objective lens on the inverted optical microscope (Leica DMI4000 B) integrated with the AFM.

The salicylic acid crystals were imaged prior to, and after, 15 min of dissolution in a solution that was undersaturated by *ca.* 16%. Since the crystals were attached on the surface, removal of solution involved pouring off the undersaturated solution, and pouring water over the crystal and blow drying with nitrogen. The water was used to avoid crystallisation of dissolved material in a quick washing process that did not contribute to the substantial pitting of the surface that occurred during the 15 min of dissolution in the undersaturated solution.

## Simulations and modelling

### Equilibria involved in crystal dissolution

Salicylic acid is a weak acid and this needs to be accounted for in the treatment of dissolution kinetics. In solution, the following equilibrium prevails: 1
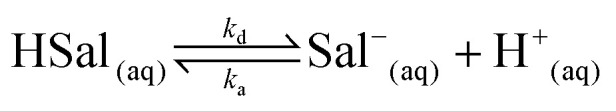
where HSal represents salicylic acid, Sal^−^ represents the salicylate ion and *k*_d_ and *k*_a_ represent the rate constants for dissociation and association, respectively.

The salicylic acid dissociation constant, *K*_a_, is 1.05 × 10^−3^ and considering *k*_a_/*k*_d_ = *K*_a_, and that *k*_a_ can reasonably be considered to be diffusion-controlled, we were able to deduce *k*_d_ from the activity corrected *K*_a_ value for use in the various simulations. Protons are reduced at the working electrode (UME) tip as follows:

2 H^+^_(aq)_ + e^−^ ⇌ 1/2H_2(g)_

These reactions, (1) and (2), are illustrated in Fig. 1c.

The reduction of protons at the electrode causes the equilibrium in eqn (1) to shift to the right, and therefore the concentration of salicylic acid (HSal) decreases. When the UME is in close proximity to the crystal surface, this undersaturation causes the crystal surface to dissolve:3
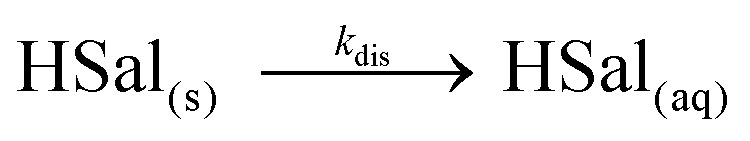
as illustrated in Fig. 1c. Thus, the crystal substrate provides a flux of HSal, *J*_HSal_, caused by a local undersaturation between the tip and crystal. The magnitude of this flux is reasonably given as a first order process in undersaturation for our purposes:

4 *J*_HSal_ = *k*_dis_(*c*_HSal_ − *c*_HSal,sat_)

where *c*_HSal_ is the concentration of salicylic acid at the crystal/solution interface, and *c*_HSal,sat_ is the concentration of salicylic acid in saturated solution,^9^ and *k*_dis_ is the dissolution rate constant, which is determined from the current response.

### Finite element method simulations

**Fig. 3:**
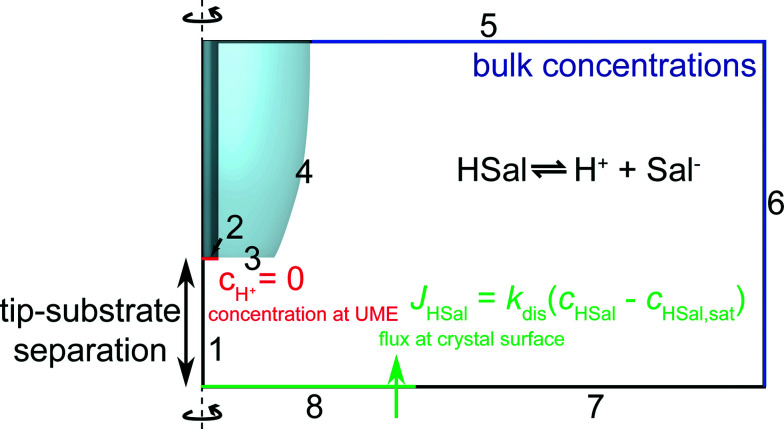
Geometry (not to scale) of the FEM model used to simulate tip current response for various values of the dissolution rate, *k*_dis_.

FEM modelling was performed using COMSOL Multiphysics 4.2a (COMSOL AB, Sweden) running on a Dell Intel core 7i Quad 2.93 GHz computer equipped with 16 GB of RAM and Windows 7 Professional ×64 bit. The basic geometry for the model is shown in Fig. 3. To maximise computational efficiency, an axisymmetric cylindrical 2-dimensional (2D) model with symmetry axis boundary 1, was built with a much finer mesh near the surfaces of the electrode and the crystal.^23,24^ Three interdependent species were modelled as defined in eqn (1). For the experimental conditions, the transport of these species was predominantly by diffusion, which was treated by solving the following equation, a form of Fick's Second Law:

5 ∇·(*D*_*j*_∇*c*_*j*_) + *R*_*j*_ = 0

where *D*_*j*_ is the diffusion coefficient, *c*_*j*_ is the concentration and *j* is the species of interest. *R*_*j*_ is a kinetic term representing the loss and/or formation of species *j* according to eqn (1) which is always at equilibrium. *D*_Sal−_ = *D*_HSal_ = 8.4 × 10^−10^ m^2^ s^−1^,^38^ and *D*_H+_ = 7.6 × 10^−9^ m^2^ s^−1^.^39^

The boundary conditions can be understood as follows. As discussed, boundary 1 represents a line of axisymmetric symmetry. On boundary 2, protons are reduced at a diffusion limited rate. Thus, the following Dirichlet boundary condition applies:

6 boundary 2: *c*_H+_ = 0,

where *c*_H+_ is the concentration of protons. For other species (Sal^−^, HSal), a no normal flux Neumann boundary condition applies, as follows:

7 boundary 2: **n**·*D*_*j*_∇*c*_*j*_ = 0

where **n** is a unit vector normal to the boundary, from the crystal surface into the solution. Boundaries 3 and 4 are glass surfaces on the electrode where all species are inert. Similarly, boundary 7 represents the glass petri dish. Note that the crystal and petri dish were set to be co-planar because with the induced dissolution mode, the crystal reaction is confined to the part of the crystal directly under the active part of the tip. Thus, no flux Neumann conditions apply:

8 boundary 3, 4 and 7: **n**·*D*_*j*_∇*c*_*j*_ = 0

Boundaries 5 and 6 represent the bulk solution and are therefore determined by the bulk concentrations of HSal, Sal^−^ and H^+^, as calculated by MINEQL^+^ (Environmental Research Software, version 4.6) which used the Davies equation to calculate activity corrected ion speciation.^40^ In these regions, a Dirichlet boundary condition is applied as follows:

9 boundary 5–6: *c*_*j*_ = *c*_bulk,*j*_

where *c*_bulk,*j*_ is the bulk concentration of species *j*. Finally, on boundary 8, a flux condition is enforced, using eqn (4), which results in a Robin boundary condition:

10 boundary 8: **n**·(*D*_HSal_∇*c*_HSal_) = −*J*_HSal_

A range of values for *k*_dis_ (eqn (4)) were input into the model. The entire tip *z*-approach curve was modelled for a particular *k*_dis_ using a parametric sweep which altered the geometry of the model by gradually reducing the separation between the UME and the crystal surface, *d*.

## Results and discussion

**Fig. 4:**
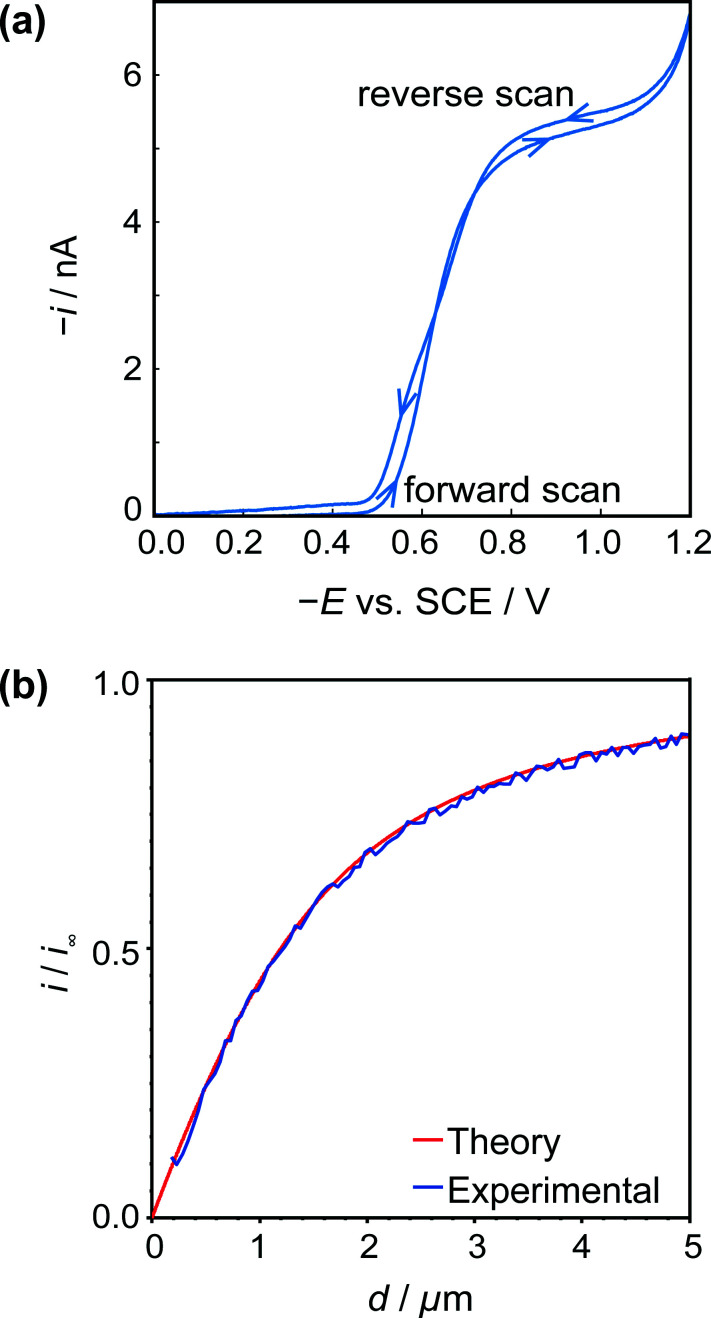
(a) Typical cyclic voltammogram for the reduction of HSal (*via* dissociation to H^+^ and Sal^−^) at a 2.5 μm diameter Pt UME. (b) Tip approach curves (applied potential −0.8 V) to an insulating (glass) surface with the same UME. The blue approach curve is the experimental data, and red curve is the theoretical result for hindered diffusion for an electrode with RG = 15.^44^

A typical CV for H^+^/HSal reduction in the solution of interest (*vide supra*) is shown in Fig. 4a, at a potential scan rate of 0.1 V s^−1^. The current attains a steady-state limiting plateau in the potential range −0.8 V to −1.1 V *vs.* SCE and at more cathodic potentials hydrogen evolution from the water is initiated, resulting in a further increase in the current magnitude. Under the experimental conditions the concentration of free protons (pH 4.5) is low and the protons essentially come from the dissociation of the weak acid in solution (eqn (1)), promoted by the removal of H^+^ at the tip UME (eqn (2)). In fact, the limiting current, *i*_∞_, is essentially controlled by the bulk HSal concentration and *D*_HSal_ value, because the dissociation of weak acids, such as HSal, is so rapid,^41^ as highlighted above. Thus,

11 *i*_∞_ = 4*FD*_HSal_ac^*^_HSal_

with *D*_HSal_ = 8.4 × 10^−6^ cm^2^ s^−1^ and c^*^_HSal_*ca.* 10 mM, this predicts *i*_∞_ = 4.0 nA, close to the experimental value.

Fig. 4b shows a typical *z*-approach curve for the UME over the glass petri dish, in which the tip was translated in the *z*-direction towards the substrate whilst the steady-state limiting tip current (at an applied potential of −0.8 V) was recorded. As the tip comes closer to the inert glass substrate, the diffusion of HSal is hindered, in a process called negative feedback.^42,43^ There is a close match of experiment and theory,^44^ and the current measured at IC (detected as a damping of the tip oscillation, as explained above) gives the distance of closest approach of 180 nm indicating good alignment between the electrode and glass substrate.

The UME current has been normalised, *i.e.* is presented as *i*/*i*_∞_, where *i* is the measured current and *i*_∞_ is the ‘bulk’ current, at an infinite distance from the surface.^35^ The ‘bulk’ current, was actually taken at *d* = 12.5 μm, where *d* is the distance between the tip electrode and the crystal, whilst *z* (*vide infra*) is the tip position defined by the piezoelectric positioner.

**Fig. 5:**
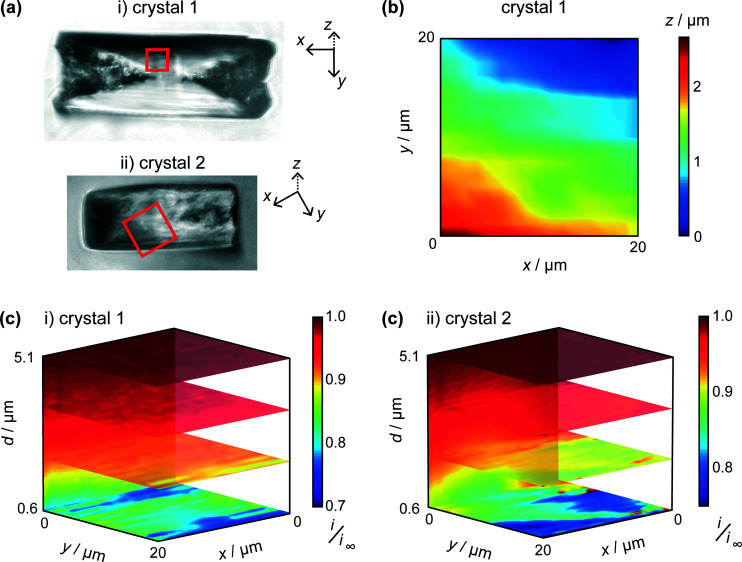
(a) Bright field optical microscopy images of i) crystal 1 and ii) crystal 2, taken after HIC-SECM. The 20 × 20 μm scan area is represented by the red square. (b) Topography of crystal 1 with the lowest point of the crystal imaged designated as *z* = 0. (c) 3D plots of the normalised tip current (*i*/*i*_∞_), revealed above crystals 1 and 2, with several horizontal and vertical slices through the data sets highlighted.

Two typical crystals (Fig. 5a) were imaged using HIC-SECM. The red squares in these optical images indicate the regions of the 20 × 20 μm scan areas in relation to the crystal surface. We will distinguish between the two crystals by naming them ‘crystal 1’ and ‘crystal 2.’

One of the advantages of using IC-SECM is that the feedback allows for us to plot the topography of the surface, *i.e. z*-position of the piezo at IC. It can be seen that this is largely manifest as a small tilt on the crystal surface, as is shown in Fig. 5b, which shows the topography for crystal 1. For each crystal, the overall tilt of the surface allowed the distance of closest approach of the UME at IC to be estimated from a simple geometrical analysis of a planar tip above a tilted surface. This was 0.6 μm for each crystal.

As discussed above, and exemplified in our recent work,^30,45^ HIC-SECM allows 3D electrochemical flux (current) data to be acquired at and above a surface. Fig. 5c shows normalised current data, presented as slices of the scan in several *x*–*y* (parallel to the crystal surface) and one *x*–*z* (perpendicular to the crystal surface) planes for the two crystals. From Fig. 5c, it can be observed that there is a drop in tip current from the bulk solution to the surface of crystals 1 and 2, typically with a value at the end of the approach curve (at IC) between 0.75 and 0.8 of the bulk current. For comparison, at this distance (0.6 μm from the surface), the current ratio would be considerably lower, 0.28 for an inert surface.^44^ The higher current indicates induced dissolution, but with rather slow kinetics.^26^

**Fig. 6:**
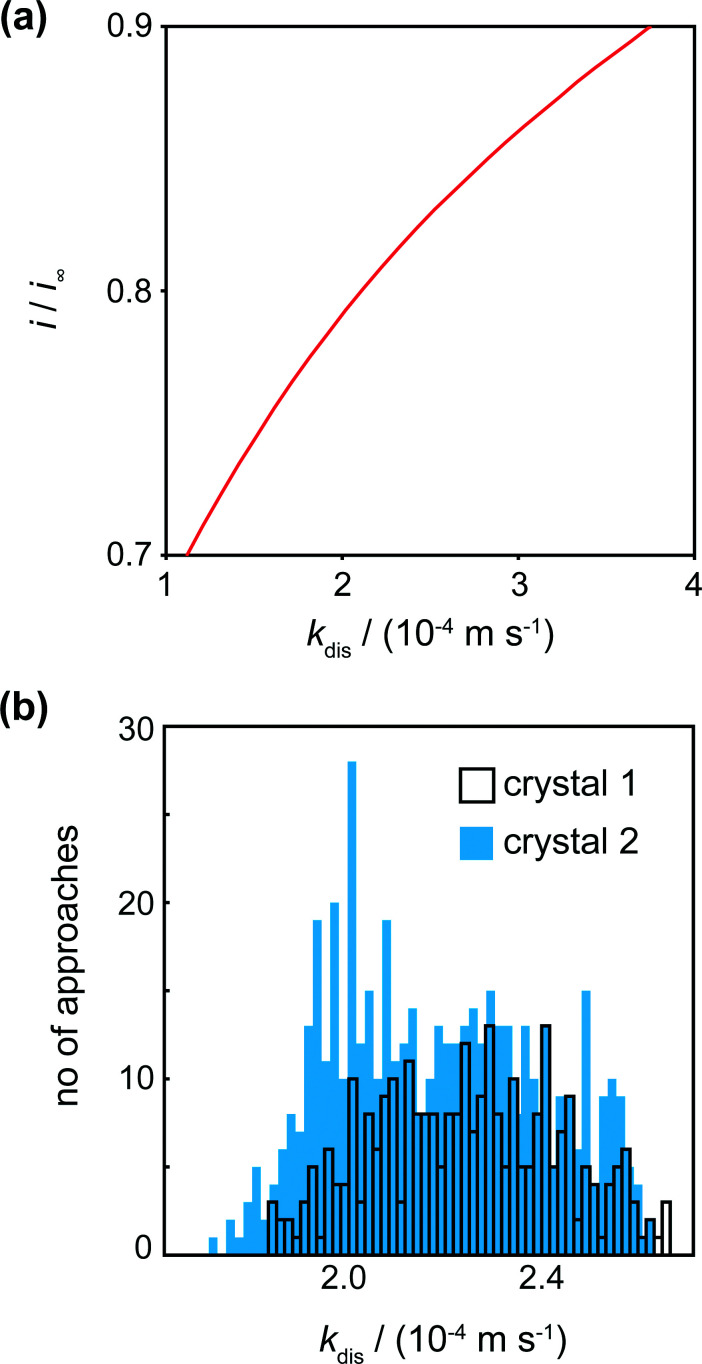
(a) Working curve of *i*/*i*_∞_*vs. k*_dis_ for *d* = 0.6 μm (distance of closest approach) enabling kinetic constants to be deduced from *i*/*i*_∞_. (b) Histograms showing the spread of *k*_dis_ values for various locations on the 2 crystals. Note that crystal 1 had fewer approaches (289) than crystal 2 (400).

The tip current at IC (the distance of closest approach) was measured and compared to simulated data for this tip position for a range of dissolution rate constants (Fig. 6a). For a range of *k*_dis_ values, at a fixed tip-to-substrate separation (180 nm, *i.e.* at the closest point in the approach curve), the COMSOL model described above was used to calculate a value for *i*/*i*_∞_. The resulting curve is the spline fit for these data. The distribution of *k*_dis_ values obtained in this way is shown in the histograms in Fig. 6b for crystal 1 and crystal 2.

For crystals 1 and 2, the dissolution rate constants determined from the tip current at the distance of closest approach, as shown in Fig. 6a, were 2.3 (±0.4) × 10^−4^ m s^−1^ and 2.2 (±0.4) × 10^−4^ m s^−1^, respectively. These values are consistent and over a rather narrow range. There is thus only a small degree of heterogeneity of dissolution rate constants across the surface.

**Fig. 7:**
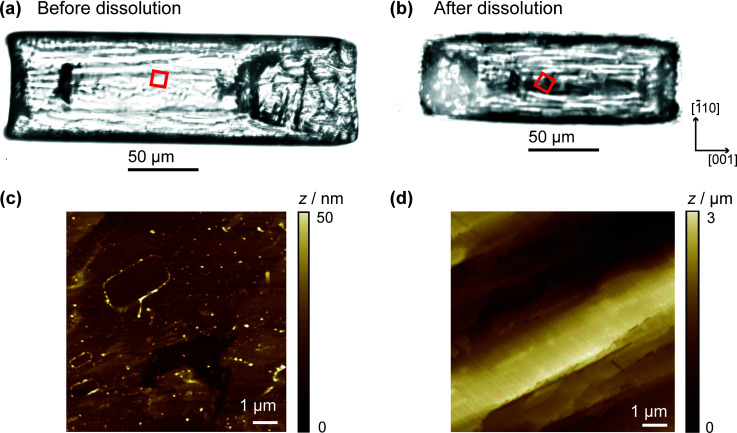
Bright field optical microscopy images of a salicylic acid crystal, not imaged by HIC-SECM, (a) before dissolution (as-grown) and (b) after 15 min dissolution in an 8.4 mM solution of salicylic acid. The 9 × 9 μm scan area is represented by the red square. (c) *Ex situ* AFM topography images of the crystals in (a) and (b) are shown in (c) and (d), respectively. Note the difference in height scale bars.

It is informative to compare the kinetics to previous measurements. In our recent work combining optical microscopy with FEM simulations and vertical scanning interferometry, the flux values for the (110) face were of the order 10^−5^ mol m^−2^ s^−1^ for solutions undersaturated by 10–20%.^9^ Even without correcting for mass transport, this gives an effective dissolution rate constant ~10^−2^ m s^−1^ which is about 50 times larger than measured herein. Still higher fluxes have been measured in other studies.^17,18^ To rationalise the differences, we used AFM to compare the crystal surface as-grown (and studied herein), and the same crystal after 15 min of ‘bulk’ dissolution in a solution that was undersaturated by *ca.* 16%. The crystals in the images shown in Fig. 7 were both washed with water prior to imaging, which may account for some of the surface features observed in Fig. 7c, but cannot account for the huge variation between the surfaces of the crystal in 7c and d. The comparison is made in Fig. 7, which shows that the (110) face of as-grown salicylic acid exhibits a relatively flat surface with a little microstructure, but after only 15 min moderate dissolution the surface roughens extensively and is covered in a very high density of step sites and etch features. These are evidently responsible for the greatly enhanced activity of earlier work.^9,17,18^

In contrast, the HIC-SECM experiments relate to the surface shown in Fig. 7c, as dissolution occurs only transiently when the UME probe encounters the crystal surface during each approach. Because dissolution is only induced momentarily, the value of dissolution kinetics relates much more closely to the intrinsic kinetics of the (110) plane of salicylic acid, which is evidently rather slow. In practical applications, there will be a significant transition in the crystal microstructure between that shown in Fig. 7a and c to that in Fig. 7b and d. This has a profound effect on dissolution and the dramatic (time) evolution in kinetics, as evidenced by the studies herein and our earlier work,^9^ needs to be taken into account when building holistic dissolution models for these types of materials.

## Conclusions

HIC-SECM has been introduced as a new quantitative approach for the measurement of dissolution kinetics. Moreover, we have generally extended SECM dissolution methodology to weak acids, an important class of materials covering many pharmaceuticals. Dissolution can be induced electrochemically by reducing free protons in solution to hydrogen, which perturbs (decreases) the concentration of undissociated acid in solution and makes the solution near the crystal locally undersaturated. The current flowing at the tip then depends, in part, on the dissolution kinetics, which can be elucidated by FEM modelling of processes in the tip/crystal gap.

An important aspect of the HIC-SECM technique is that the UME probe only induces dissolution when in close proximity to the crystal surface. Thus, by hopping the tip to and from the crystal, to build up a scan, dissolution is only induced transiently and the crystal is studied in a state close to ‘as-grown.’ We have shown that the dissolution kinetics at such a surface is much slower than for a crystal which has undergone more extensive dissolution. By carrying out AFM measurements on ‘as-grown’ crystals and those which have been subjected to dissolution, this difference in activity has been rationalised as being due to a significant change in the surface morphology: the ‘as-grown’ (011) surface studied has comparatively little microstructure and nanostructure and is characterised by extended terraces, whereas even after moderate dissolution, the surface becomes covered in an abundance of steps and etch features which promote dissolution. This type of transition evidently has a massive impact on dissolution kinetics and we will report on other pharmaceutical crystal systems in due course.
